# Changes in adolescents’ and parents’ intakes of sugar-sweetened beverages, fruit and vegetables after 20 months: results from the HEIA study – a comprehensive, multi-component school-based randomized trial

**DOI:** 10.3402/fnr.v59.25932

**Published:** 2015-03-20

**Authors:** Mona Bjelland, Solveig E. S. Hausken, Ingunn H. Bergh, May Grydeland, Knut-Inge Klepp, Lene F. Andersen, Torunn H. Totland, Nanna Lien

**Affiliations:** 1 Department of Nutrition, Faculty of Medicine, University of Oslo, Oslo, Norway; 2 Department of Coaching and Psychology, Norwegian School of Sport Sciences, Oslo, Norway; 3 Department of Sports Medicine, Norwegian School of Sport Sciences, Oslo, Norway

**Keywords:** children, parent, fruit, vegetables, beverages

## Abstract

**Background:**

Interventions conducted in school-aged children often involve parents, but few studies have reported effects on parents’ own behaviour as a result of these interventions.

**Objective:**

To determine if a multi-component, cluster randomized controlled trial targeting 11–13 year olds influenced their consumption of fruit, vegetables, sugar-sweetened soft drinks and fruit drinks, and to explore whether the results varied by gender, adolescent weight status or parental educational level. A final aim was to assess whether the parents’ intakes were affected by the intervention.

**Design:**

Participants were 1,418 adolescents, 849 mothers and 680 fathers. Baseline and post-intervention data from the 20 months intervention study HEIA (HEalth In Adolescents) were included. Data were collected assessing frequency (and amounts; beverages only).

**Results:**

No significant differences were found at baseline between the intervention and control groups, except for the parental groups (educational level and intakes). At post-intervention, the adolescents in the intervention group consumed fruit more frequently (*P*<0.001) and had a lower intake of sugar-sweetened fruit drinks compared to the control group (*P*=0.02). The parental educational level moderated the effect on intake of sugar-sweetened fruit drinks in adolescents. The intake was less frequent in the intervention groups compared to the control groups (*P*=0.02) for those who had parents with low and medium educational level. Furthermore, the intervention may have affected mothers’ fruit intake and the vegetable intake in higher educated fathers.

**Conclusion:**

Favourable effects in favour of the intervention group were found for intake of fruit and sugar-sweetened fruit drinks among the adolescents in the HEIA study. Our results indicate that it is possible to reduce adolescents’ intake of sugar-sweetened fruit drinks across parental education, and potentially affect sub-groups of parents.

Weight gain, overweight and obesity have been associated with various dietary behaviours, as well as food choices and intake of individual nutrients/calories. Examples are diets high in fat and carbohydrates, diets low in fibre, frequency and composition of breakfast, frequent snacking and consumption of sugar-sweetened beverages (SSB) (1–5). Recent reviews conclude that intake of SSB increases the risk of obesity (6, 7), while there is possible evidence that an increased consumption of vegetables and fruit may prevent body weight gain (8–10). Previous Norwegian studies have reported a low intake of fruit and vegetables in children and adolescents (11–14). The intake of fruit and vegetables decreased among adolescents of parents with lower educational level, but increased among adolescents of parents with higher education among Norwegian 11–13 year olds in the period 2001–2008 (15). Furthermore, earlier Norwegian studies have observed a high intake of energy from added sugar and SSB (such as carbonated soft drinks and/or sugar-sweetened fruit drinks) (16), although a slight decrease was observed for frequency of intake of SSB among Norwegian 11–13 year olds in the period 2001–2008 (17).

Kremers et al. (18) argue that tests of effect modifiers should become common practice in behavioural research to increase the understanding of mechanisms of behaviour change and to optimize interventions. Gender is the most frequently studied potential moderator of school-based interventions aimed at energy balance-related behaviours, in addition to ethnicity, age, baseline values of outcomes, initial weight status and socioeconomic status (SES) (18, 19). SES has shown to be associated with poorer diets in adolescents (20) and parental education has been found to be associated with consumption of fruit, vegetables and SSB in adolescents (2, 14, 21).

A number of interventions have aimed to increase the consumption of fruit and vegetables and to reduce the intake of SSB in school-aged children (22–29). The interventions often involve parents, but to our knowledge only a few studies have reported effects on the parents’ own dietary intake as a result of these interventions (30–32).

Schools are often used as a setting for implementing interventions developed to reduce the prevalence of obesity in children and adolescents, because it offers continued and intensive contact with a large population across ethnic and socio-economic groups (33, 34). A Norwegian comprehensive, multi-component school-based randomized trial was conducted in 2006–2009. The overall goal of the HEalth In Adolescents (HEIA) study was to design, implement and evaluate a comprehensive intervention program to promote healthy weight development among young adolescent school children (11–13 year olds). The targeted changes in behaviours were to decrease consumption of SSB and sedentary behaviour, and to increase physical activity and the consumption of fruit and vegetables (35). Previous findings from the baseline data within the HEIA study showed that the intake of SSB was higher during weekend days (means; girls 2.1/boys 2.5 dl per day) than during weekdays (means; girls 1.0/boys 1.4 dl per day), whereas the frequencies of the fruit (means; girls 1.5/boys 1.3 times per day) and vegetable intake were low (both genders; below 1.0 time per day for both raw and cooked vegetables). Significant differences were found in the adolescents’ intake of SSB and in the perceived availability of fruit, vegetables and SSB by parental education (14).

The aim of this paper was three-fold. Firstly, to determine if a multi-component health promotion intervention targeting 11–13 year olds influenced their consumption of fruit, vegetables and SSB. Secondly, the aim was to explore whether the results varied by gender, adolescent initial weight status or by parental educational level. Thirdly, the aim was to assess whether the parents’ intakes of fruit, vegetables and SSB were affected by the intervention.

## Methods

### Study design and subjects

The participants were recruited from schools located in the south-eastern part of Norway with more than 40 pupils in 6th grade. Such schools are mainly located in towns/municipalities, and 37 schools were recruited from the largest towns/municipalities in seven counties surrounding Oslo (35). All 6th graders in these 37 schools (*n*=2,165) and their parents/legal guardians were invited to take part in the HEIA study. Of these, 1,580 returned a parent signed informed consent form for the adolescent. A cluster randomized controlled pre–post study design was used to evaluate the effectiveness of the intervention; 12 schools were randomly assigned by simple drawing to the intervention group and 25 to the control group. The baseline and the post-intervention data collections took place in September 2007 and May 2009, respectively.

Power calculations were made based on changes in body mass index (BMI), intake of fruit, vegetables and soft drinks, and physical activity measured by accelerometers. Taking the cluster effect of randomly assigning schools to intervention and control into account, assuming that 80% of the pupils would participate, that the attrition rate would not exceed 15% per year, we aimed for 40 schools (10 intervention and 30 control) with an average of 45 pupils participating from each school. In the final study, we had 37 schools and the initial participation rate was 72.9% among adolescents (*n*=1,580). In total, 1,210 mothers (76.6% of 1,580) and 1,067 fathers (67.5% of 1,580) participated at baseline (35).

The adolescents and parents who participated in both the data assessments were included in this paper. A total of 1,418 adolescents (89.7% of those 1,580 returning consent), 849 mothers (53.7% of 1,580) and 680 fathers (43.0% of 1,580) were included in the analyses. Reasons for adolescents and parents not participating at the post-intervention were sickness, holiday or withdrawing from the study. The multi-component approach in the HEIA study included collaboration with school principals and teachers, school-health services and parent committees, while schoolteachers were the key persons to implement the intervention components. The intervention program consisted of a mixture of individual, group and environmental level strategies and activities (for details (35, 36)). Ethical approval and research clearance was obtained from the Regional Committees for Medical Research Ethics and the Norwegian Social Science Data Service.

### Questionnaire data

The Internet-based child questionnaire comprised mostly questions with pre-coded answer categories and could be completed at the school in about 45 min. The parental questionnaires, one for each parent (paper–pencil format), were sent home with the adolescent at both time points and completed by the parents, returned to the teachers in a sealed envelope and collected from the schools by project staff.

### Behavioural outcomes for adolescents and parents

The intakes of sugar-sweetened soft drinks and sugar-sweetened fruit drinks were assessed by frequency (six categories, from never/seldom to every weekday) and amount (in glasses, four categories: from one to four glasses or more) for weekdays and by amount for weekends (in glasses, eight categories: from never/seldom to seven glasses or more). Intake of fruit was assessed by one question and intake of vegetables was assessed by two questions (raw and cooked vegetables), asking for frequency of usual intake. Frequencies for intake of fruit and vegetables were measured using eight categories: from never/seldom to three or more times daily.

### Weight status and parental education

The age and gender specific BMI cut-off values proposed by the International Obesity Task Force (37) were used to categorize the adolescents as normal weight (including underweight) and overweight (including obese). The adolescents’ anthropometrics were assessed by trained staff, while parental height and weight were self-reported. Details of the anthropometrics of the participants and test–retest values of the measures have been reported elsewhere (35, 38). Parental education was collected as part of the informed consent form for the adolescents filled in by parents. Education was categorized into three levels: ≤12 years, between 13 and 16 years and >16 years. Data from the parent with the longest education was used in the analyses, or else the one available.

### Data analysis

Clustering effects due to schools being the unit of recruitment were checked by the Linear Mixed Model procedure. Only 0.1–3% of the unexplained variance in the adolescents’ dietary behaviours was on group level, and it was therefore decided to not account for clustering of schools.

The characteristics at baseline are presented as proportions (demographic variables), means and standard deviations (SD) (behavioural variables). Continuous variables were tested for differences between the intervention group and the control group with independent sample *t*-tests, and Chi-square test of proportions was used for categorical variables.

**Fig. 1:**
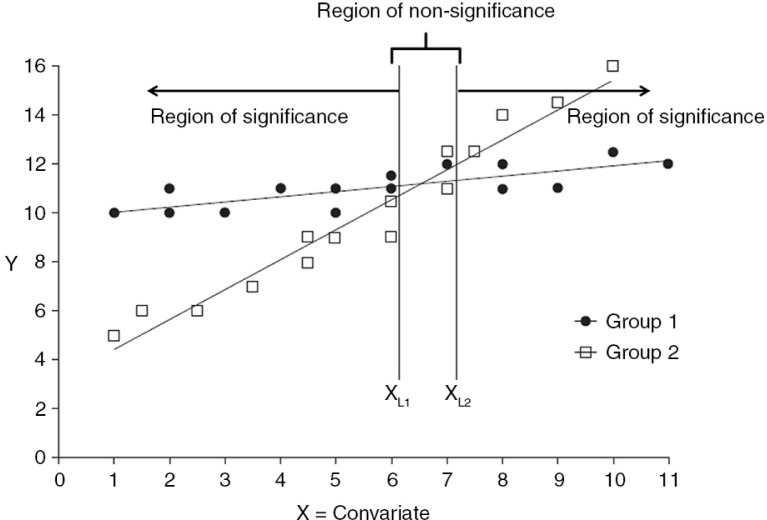
The Johnson-Neyman technique. When heterogeneous regression slopes are present this implies that the magnitude of the intervention effect (Y) is not the same at different levels of X (covariate; the baseline intake in these analyses). The Johnson-Neyman approach provides values on X associated with non-significant/significant effects, giving regions of non-significance and significance. X_L1_ is the lowest value and X_L2_ is the highest value of the non-significance region.

The effect of the intervention was determined using one-way ANCOVA with the post-intervention value for the outcomes as the dependent variables, the condition (the experimental group) as the independent variable and the baseline values of the outcomes as covariates. For parents, the ANCOVA analyses were adjusted for educational level due to a significant difference between the intervention and control group at baseline. The data were checked to ensure that there were no violations of the assumptions. Interaction effects in adolescents’ intakes by gender, initial weight status and parental educational level were tested in separate analyses as a second step, using two-way ANCOVA. For parents, heterogeneous regression slopes were found for three behaviours (intake of vegetables and soft drinks for mothers, and vegetables for fathers). When heterogeneous regression slopes are present, this implies that the magnitude of the intervention effect is not the same at different levels of X (the baseline intake in these analyses). Values on X associated with non-significant/significant effects, giving regions of non-significance and significance, is provided by the Johnson–Neyman approach (Fig. 1). The Johnson–Neyman technique is the strongest alternative to ANCOVA in experimental designs when the assumption of homogeneity of regression slopes has been violated (39). The basic difference between ANCOVA and the Johnson–Neyman approach is that the effects are estimated at the grand covariate mean with ANCOVA, but with the Johnson–Neyman technique the effects are estimated as a function of the covariate score. The Johnson–Neyman technique was used to analyse the heterogeneous regression cases (40), and a ‘Quick Johnson–Neyman Procedure Calculator’ was used to calculate the regions of non-significance and significance for separate educational levels (http://www2.gsu.edu/~epstco/). The regions of significance may lie outside the range of the covariate scores included in the sample, resulting in no region of significance within the range of sample data (40).

The significance level was set at *P*<0.05 for all analyses, except for the interaction tests where *P*<0.10 was used. Data were analysed using SPSS Statistics, version 20 (IBM Corporation, New York, USA).

## Results

Table 1 shows the baseline characteristics for the adolescents in the control and the intervention groups. No significant differences in terms of demographic and behavioural characteristics were found between the intervention and control groups. However, for the mothers and fathers there were significant differences with regards to the educational level (both mothers and fathers), intake of sugar-sweetened fruit drinks (mothers) and intake of soft drinks and vegetables (fathers) (Table 2).

**Table 1:** Adolescent baseline characteristics (demographic and behaviour) for the control and the intervention group in the HEIA study

	Control		Intervention		*P*

	*n*†=898		*n*†=498		
Gender					0.51
Boys (%)	52.2		50.4		
Girls (%)	47.8		49.6		
Weight status					0.10
Normal weight (%)	85.5		88.6		
Overweight (%)	14.5		11.4		
Parental educational level					0.15
≤12 years (%)	31.1		26.2		
13–16 years (%)	35.8		37.7		
>16 years (%)	31.1		36.1		
	Mean	SD	Mean	SD	*P*
Age (mean (SD))	11.2	0.3	11.2	0.3	0.38
Soft drinks, dl/week	5.3	5.9	4.9	5.4	0.19
Sugar-sweetened fruit drinks, dl/week	5.6	7.6	5.4	7.3	0.67
Fruit intake, times/week	9.8	6.9	9.8	7.0	0.92
Vegetables, times/week	11.1	9.5	10.9	8.6	0.65

*P*=Pearson Chi-Square and *t*-test.

† *n*=vary slightly.

**Table 2:** Parental baseline characteristics (demographic and behaviour) for the control and the intervention group in the HEIA study, female and male

	FEMALE				*P*	MALE				*P*
	
	Control		Intervention			Control		Intervention		
	
	*n*†=603		*n*†=246			*n*†=474		*n*†=199		
Weight status					0.12					0.37
Normal weight (%)	69.8		75.6			43.0		46.8		
Overweight (%)	30.2		24.4			57.0		53.2		
Educational level					**0.04**					**0.02**
<12 years (%)	38.2		28.9			37.4		25.7		
13–16 years (%)	36.7		42.6			31.1		38.8		
>16 years (%)	25.1		28.5			31.5		35.5		
	Mean	SD	Mean	SD	*P*	Mean	SD	Mean	SD	*P*
Age	41.0	4.7	41.6	4.6	0.85	43.3	5.1	44.0	5.5	0.45
Soft drink, dl/week	1.9	5.0	1.8	4.3	0.67	4.4	7.7	3.3	6.3	**0.02**
Sugar-sweetened fruit drinks, dl/week	1.3	4.0	2.2	4.9	**<0.001**	1.9	4.4	2.0	4.4	0.90
Fruit intake times/week	8.2	5.5	8.2	5.3	0.48	5.6	4.8	5.9	5.0	0.47
Vegetables times/week	10.3	5.8	10.8	5.6	0.57	7.2	4.9	8.0	5.7	**0.03**

*P*=Pearson Chi-Square and *t*-test.

† *n*=vary slightly.

P-values in bold indicate significant values.

**Fig. 2:**
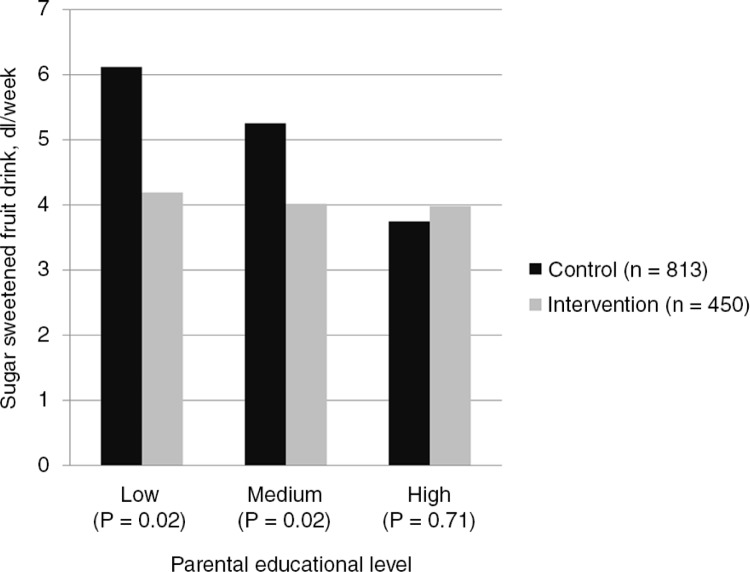
Effect at 20 months assessment of the HEIA study, total sample of adolescents (adjusted for baseline).

Significant differences were found for the adolescents at the post-intervention assessment between the intervention group and the control group in fruit consumption (*P*<0.001), and intake of sugar-sweetened fruit drinks (*P*=0.02) (Table 3). The intervention group consumed fruit more frequently, and had a lower intake of sugar-sweetened fruit drinks compared to the control group after the intervention. Analyses of moderating effects by adolescents’ gender, initial weight status and parental education revealed an interaction for parental educational level and intake of sugar-sweetened fruit drinks only (*P*=0.06). Stratified analyses showed that the total amount of sugar-sweetened fruit drinks consumed was lower for the intervention groups compared to the control groups after the intervention (*P*=0.02) for those who had parents with low and medium educational level (Fig. 2). There were no significant differences in the total amount of sugar-sweetened fruit drinks consumed for those who had parents with a high educational level.

**Table 3:** Effects at 20 months assessment of the adolescents in the HEIA study, total sample

	Control		Intervention		*P*

	Mean^a^	Cl	Mean^a^	Cl	

	*n*†=896		*n*†=502		
Soft drinks, dl/week	6.0	(5.6, 6.5)	6.3	(5.8, 6.9)	0.41
Group×gender					0.23
Group×WS					0.89
Group×PE					0.52
Fruit drinks, dl/week	5.1	(4.7, 5.6)	4.2	(3.6, 4.8)	**0.02**
Group×gender					0.64
Group×WS					0.99
Group×PE					0.06
Fruit intake, times/week	9.6	(9.1, 10.0)	10.9	(10.4, 11.5)	**<0.001**
Group×gender					0.64
Group×WS					0.32
Group×PE					0.92
Vegetables, times/week	10.5	(10.0, 11.1)	10.9	(10.1, 11.6)	0.46
Group×gender					0.56
Group×WS					0.26
Group×PE					0.22

Group=intervention and control; WS=weight status; PE=parental educational level. Fruit drinks: sugar-sweetened fruit drinks. Analyses: overall for all, one-way ANCOVA. Group×WS/group×PE: separate interaction analyses for weight status and for parental education, two-way ANCOVA.

a Adjusted for baseline.

† *n*=vary slightly. P-values in bold indicate significant values.

For the parents, a borderline significant difference in maternal intake of fruit (*P*=0.06) was found at post-intervention, indicating a higher intake in the intervention group (Table 4). By use of the Johnson–Neyman technique, we were able to identify regions of non-significance by educational level for soft drink intake in the highest educated mothers, and vegetables intake for the medium and highest educated fathers (Table 4). There was no significant difference when the intake of soft drinks among the highest educated mothers was lower than 0.7 dl per week at baseline. When the intake at baseline was above 0.7 dl per week, the intervention group had a higher intake of soft drinks at post-intervention compared to the control group. The pattern was the same for the vegetable intake among the highest educated fathers. No significant difference was found when the intake was less frequent than 13.2 times per week at baseline, but when the intake was above 13.2 times per week, the intervention group ate vegetables more frequently at post-intervention compared to the control group. Finally, for intake of vegetables in fathers with 13–16 years of education, the region of non-significance was between 2.0 and 10.1 times per week. When the intake was lower than 2.0 times per week at baseline, the post-intervention intake was higher in the control group compared to the intervention group – meaning no effect on the low consumers in the intervention group. However, when the baseline intake was above 10.1 times per week, the intervention group ate vegetables more frequently at post-intervention compared to the control group. No interpretation was possible for the other behaviours (consumption of vegetables in mothers and low educated fathers, and intake of soft drinks in mothers with low and medium educational level), as the regions of significance may lie outside the range of covariate scores included in the sample (40).

**Table 4:** Effects at 20 months assessment of the parents in the HEIA study

	Female				*P*	Male				*P*
	
	Control		Intervention			Control		Intervention		
	
	Mean	Cl	Mean	Cl		Mean	Cl	Mean	Cl	
	
	*n*†=603		*n*†=246			*n*†=479		*n*†=201		
Soft drinks, dl/week	–	–	–	–	JN	3.9	3.5, 4.4	3.8	3.0, 4.5	0.72
Sugar-sweetened fruit drinks, dl/week	1.4	1.2, 1.7	1.2	0.9, 1.6	0.32	1.7	1.3, 2.1	1.9	1.3, 2.4	0.62
Fruit intake, times/week	8.4	8.1, 8.8	9.1	8.5, 9.7	0.06	5.9	5.5, 6.2	6.0	5.4, 6.6	0.70
Vegetables, times/week	–	–	–	–	JN	–	–	–	–	JN

The Johnson–Neyman technique Regions of non-significance^a^										

	Female					Male				

Soft drinks, dl/week	Low value			High value		Low value			High value	
Highest education	n.a.			0.7						
Vegetables, times/week										
Highest education						n.a.			13.2	
Medium education						2.0			10.1	

JN=The Johnson–Neyman technique; n.a.=not applicable.

† *n*=vary slightly.

a For the other levels of education; the regions of significance were outside the range of the covariate scores included in the sample, resulting in no region of significance within the range of sample data. The same was the case for female intake of vegetables.

## Discussion

Favourable effects in favour of the intervention group were found for intake of fruit and sugar-sweetened fruit drinks among the adolescents in the HEIA study. Children of parents with low and medium educational level reduced their intake of sugar-sweetened fruit drinks the most. For parents, a borderline significant difference in maternal intake of fruit was found at post-intervention, indicating a higher intake in the intervention group. By use of the Johnson–Neyman technique, we found that higher educated fathers in the intervention group had a higher intake of vegetables at post-intervention compared to the control group, when their baseline intake was high. When the higher educated mothers’ intake of soft drinks at baseline was above 0.7 dl per week, we found that the intervention group had a higher consumption of soft drinks at post-intervention compared to the control group.

The HEIA study turned out to have a favourable effect on the consumption of fruit, but not on the intake of vegetables. In general, the preference is higher for fruit compared to vegetables (41). Additionally, fruit is more practical to eat all day long as between meal snacks compared to vegetables (42). Moreover, the typical meal pattern in Norway is one hot meal (dinner) and two or three cold meals (43), and the cold meals usually contain bread or cereals. Traditionally, vegetables are mostly eaten at dinner (44). This may explain why the HEIA intervention succeeded in increasing the adolescents’ fruit intake only. The results suggest that future interventions should focus on increasing preferences for and the frequency of intake of vegetables (41).

Awareness of health information and knowledge to choose and initiate healthy behaviours to form healthy lifestyles has been related to education in previous studies (45–47). Parental education seems to be an important factor, and it has been suggested that better educated people have the necessary health information, skills, knowledge, values and psychological control needed to choose and initiate healthy behaviours to form healthy lifestyles (48). Associations between nutrition knowledge and eating behaviour have been reported both for adults and adolescents (49, 50), and significant differences in knowledge between socio-demographic groups have been found. Men have poorer knowledge than women, and knowledge decline with lower educational level and socio-economic status (51). The HEIA study may have contributed to higher awareness/nutrition knowledge of the sugar content of sugar-sweetened fruit drinks in lower educated parents, explaining why children of parents with low and medium educational level reduced the intake of sugar-sweetened fruit drinks the most. Additionally, the adolescents having parents with the low and medium educational level had the largest potential for reducing their intake of sugar-sweetened fruit drinks.

When comparing the results from our study with other intervention studies aimed at reducing the consumption of SSB among children/adolescents, only two of the six identified studies reported effects by gender, while none reported effect on SSB intake by weight status or parental education (24–29). Subgroup analyses are called for by some and criticized by others (52). Within the HEIA project we have contributed with new knowledge by exploring whether the effects varied by sub-groups (gender, adolescent initial weight status and by parental educational level) (36, 53–55). Our findings suggest that subgroup analyses are important for being able to identify specific groups benefiting from the intervention and/or giving hints about effective components within the intervention program (52).

Finally, the HEIA intervention may have affected mothers’ fruit intake and the vegetable intake in higher educated fathers. Results from the Pro Children intervention showed no effect of the intervention on mothers’ fruit and vegetable intake at 1 year and 2 year follow-ups (30). The High 5 Intervention resulted in higher consumption of fruit and vegetable among parents in the intervention group compared to the control group at first follow-up (12 months), but the effect was not maintained at the second follow-up (1 year later) (32). Finally, positive results were reported in a short-term intervention comparing a social marketing campaign to a 5-a-Day curriculum-only intervention, and to no intervention, on increasing fruit and vegetable consumption. The intervention increased the number of servings of fruit and/or vegetables consumed by parents at post-test (after 4 weeks intervention), compared to the pre-test (31). The results from these school-based interventions may suggest that parents are affected and can benefit from dietary interventions targeting their children.

The ‘boomerang effect’, meaning engendering effects opposite to the intended ones (56), found for the mothers’ intake of soft drinks in the intervention group within the HEIA study may be the result of one or a combination of several factors. Reactance may be one reason, defined as the state of being aroused in opposition to a perceived threat to personal choice (56). Bushman (57) found that by warning people about the harmful effect of fatty products, this made them want to eat more fatty products, and this could have been the case for the mothers in the HEIA study as well. Another reason may be ‘counter-norm communications’; by describing the norms in order to argue against them, the sender may add to the receiver's knowledge of the norms and, thereby, heighten the amount of soft drinks found acceptable. A study on information gain by Greenberg (58) indicates that this may be true on some occasions. A third explanation of the finding may be what Harnack et al. (59) call ‘an intervention-related bias in food reporting’; in our study this means that the mothers in the intervention group became more aware of their intake during the intervention and therefore reported more exactly the amount consumed compared to the control group. Finally, it could also just be a consequence of randomness.

### Limitations and strengths

Our research has some limitations. The sample was recruited from a limited geographic area in south-eastern Norway, and this may limit the potential for generalization of the findings. The recruitment of schools and participants may have caused a sampling bias, restricting the number of overweight/obese participants and resulting in reduced precision (larger confidence intervals). Furthermore, the SSB consumption variables have not been validated, but our results are in line with data from a national representative study of adolescents (60). However, the main limitation is that the Johnson–Neyman technique was not applicable for all the parental outcomes. Finally, some degree of social desirability may be present in the data (61). One of the strengths of the present study is the large sample of parents, with both mothers and fathers included. Another strength is that parental education was reported by the parents themselves and that we were able to collect these data from nearly all the parents giving their adolescent consent to participate in the study, and not only from those parents answering a questionnaire. Finally, the participation rate for the adolescents with consent was high, with 90% participating at both baseline and post-intervention.

## Conclusions

The HEIA intervention increased the adolescents’ intake of fruit and decreased their intake of sugar-sweetened fruit drinks, while no increase in the consumption of vegetables was detected. Children of parents with low and medium educational level reduced their intake of sugar-sweetened fruit drinks the most, reducing social inequality in intake of sugar-sweetened fruit drinks. More research is needed to find strategies for how to increase intake of vegetables in Norwegian school children. Furthermore, the intervention may have affected mothers’ fruit intake and the vegetable intake in higher educated fathers. In a public health perspective, our results indicate one main challenge, that is, how to improve intake of vegetables both among children and their parents.
